# Factor structure and measurement invariance of the psychosocial risk factors inventory of NOM-035-STPS-2018

**DOI:** 10.3389/fpsyg.2022.1022707

**Published:** 2023-01-04

**Authors:** Julio César Cano-Gutierrez, Juan Carlos Pérez-Morán, Brando Bernal-Baldenebro, Daniela Arenas-Meneses, Ramsés Vazquez-Lira, Jesús Everardo Olguín-Tiznado

**Affiliations:** ^1^Faculty of Engineering, Architecture and Design, Autonomous University of Baja California, Ensenada, Mexico; ^2^Promoter Network of Diagnostic Evaluation Methods and Educational Innovation, Mexico City, Mexico; ^3^Institute for Educational Research and Development, Autonomous University of Baja California, Ensenada, Mexico; ^4^Faculty of Psychology, National Autonomous University of Mexico, Mexico City, Mexico

**Keywords:** invariance, sectors of activity, factor structure, validity, psychosocial risk factor, industrial sector, confirmatory factor analysis

## Abstract

The primary objective of this study was to analyze the psychometric properties of the *Inventory for the Identification and Analysis of Psychosocial Risk Factors* (IIA-PRF) of Reference Guide III proposed in NOM-035-STPS-2018. A total of 2,149 workers in Baja California, Mexico’s *industrial* and *education-government* sectors, were administered an online inventory version. Preliminary analyses were performed, as well as a Confirmatory Factor Analysis (CFA) based on two models proposed by the standard itself: an eight-factor model (8-FM) and a four-factor model (4-FM). Likewise, based on the results and with the recommendations of the specialists, a threefactor model (3-FM) was proposed. In addition, nested model sequencing methods were subsequently applied to validate the invariance between the origin of the activity. The dimensionality of 3-FM was found to have adequate fit values according to a-priori established criteria. It is concluded that the IIAPRF does not have the reliability and validity parameters necessary to support interpretations, uses and consequences based on the theoretical structure established by NOM-035-STPS-2018 and that, although the 3-FM presents better reliability and validity indices, it is not invariant in terms of the origin of the activity. Finally, we discuss the implications and recommend reviewing and adjusting the design of the IIAPRF items to extend the measurement of invariance to other groups of relevance for decision making in the improvement of the work environment.

## Introduction

Psychosocial Risk Factors (PRF) at work have been a topic of great interest for researchers and professionals in the field of occupational health for more than 40 years (Cassel, 1974; la Rocco and Jones, 1978; Van Dijkhuizen and Reiche, 1980; Hirschfeld and Cross, 1982; Shipley, 1987; Woods and Carlyle, 2002; Kompier and Taris, 2005; Houdmont and Leka, 2010; García et al., 2016; De Sio et al., 2018; Dahler-Larsen et al., 2020; Méndez and Mul, 2021). In the workplace, interest in the study of PRF is associated with the need to solve psychosocial problems (e.g., occupational stress, burnout, and harassment, among other occupational hazards) faced by workers. These factors generate deterioration in labor relations and organizational climate and an increase in sick leave, absenteeism, health insurance costs, and disability due to health problems (Rubio et al., 2010; Chirico, 2017; Fløvik et al., 2019; Salamanca et al., 2019; Van der Molen et al., 2020; Llorca-Pellicer et al., 2021; Sala et al., 2021; Standal et al., 2021).

Organizations such as the International Labor Organization (ILO), the World Health Organization (WHO), the European Agency for Safety and Health at Work (EU-OSHA), and the National Institute for Occupational Safety and Health (NIOSH) have been promoting the study and evaluation of PRF in workplaces in various economic and social sectors for four decades (Comité Mixto OIT-OMS, 1984; European Agency for Safety and Health at Work, 2000; Murphy, 2002). Moreover, authors such as Cox and Griffiths (2005) have found that the complexity of PRF for their evaluation, prevention, and intervention contributes to the generation of errors in decision-making in organizations, consequently leading to psychosocial risks at work. Among the negative effects reported by these authors are the inadequate design and management of work, poor attention to the organizational climate causing damage to the work environment, and psychological and physical problems in the worker. Several instruments have been designed in different parts of the world to evaluate the PRF at work by the ILO declaration (Dicke et al., 2018; Burr et al., 2019; Martinez-Gutierrez and Cruz-Ibarra, 2020; Žutautinė et al., 2020). For example, in European countries, instruments have been developed that systematically evaluate the PRF to promote the continuous improvement of safety in the workplace (Kristensen et al., 2005; Pejtersen et al., 2010; Ferrer et al., 2011; Burr et al., 2019; Valdiviezo et al., 2019; Gyllensten et al., 2020; Hjorthen et al., 2020). In the Latin American context, there are few examples of consolidated instruments for evaluating PRF (Salamanca et al., 2019). Among the best-known instruments are: the *Questionnaire for the evaluation of Psychosocial Factors* (CEFAP, by acronyms in Spanish), which is applied in Argentina (Ferrari et al., 2016); the *Trade Union Institute for Labor, Environment and Health 21* (ISTAS-21, it is by acronyms in Spanish) which is applied in Chile and is an adapted version in the Spanish language of the *Copenhagen Psychosocial Questionnaire* (COPSOQ; Moncada et al., 2005; Alvarado et al., 2012); the *Battery of Instruments for the Assessment of Psychosocial Risk Factors* developed by the Colombian Ministry of Social Protection (MPS) in 2010 (Ministerio de la Protección Social, 2010); and the *Psychosocial Factors in the Workplace Questionnaire* (CFPT, it is by acronyms in Spanish), designed by Dr. Noemí Silva in Perú as a questionnaire for the evaluation of PRF in the workplace (Pando et al., 2016).

The COPSOQ was designed to measure the social and psychological characteristics of jobs. Its original version presents 30 scales (Kristensen et al., 2005). The scales analyzed in these instruments have similarities and some particularities. More recent studies have grouped the items in five scales: (a) *Demands* which refers to quantitative demands, work pace, cognitive demands, emotional demands, and work influence; (b) *Influence and development,* which relates to work influence, development possibilities, control over work and work meaning; (c) *Interpersonal relationships and leadership* which refers to role clarity, role conflict, leadership quality, supervisor’s social support and sense of belonging at work; (d) *Job insecurity* is about working conditions, work environment and job insecurity; and (e) *Strain—effects and outcomes* which refers to the intention to quit, job satisfaction, general health, energy, and mental well-being and fatigue. The following factors are analyzed alpha estimates were all above the usual *α* ≥ 0.7 criteria and Root Mean Square Error of Approximation (RMSEA) = 0.039, Comparative Fit Index (CFI) = 0.947, Normed Fit Index (NFI) = 0.903, and Minimum Discrepancy Function by Degrees of Freedom divided (CMIN/DF) = 2.085 (Useche et al., 2019).

Other instruments are used in Europe to measure FRP. Among them is the DECORE Questionnaire for the Psychosocial Risk Assessment, which consists of four scales: (a) *Cognitive demands* refers to the requirements that workers must meet, which are related to the perception of the quantitative workload that the worker carries out, (b) *Control* this scale assesses the possibilities that the worker has so that he can determine the methods of organization of work and the decisions related to the organization of work activities, (c) *Organizational support* assesses the perception of the workers about the relationships that they have with colleagues and supervisors, and (d) *Rewards* this scale assesses the benefits that the worker perceives due to the performance of his/her tasks (especially it assesses the perception of salary and job safety). The coefficients Cronbach’s alpha ranged between 0.81 and 0.84, and the fit indices CFI, TLI, and SRMR for the original model to the four factors (cognitive demands, control, organizational support, and rewards) were below acceptable values (0.82, 0.80, and 0.12, respectively). Only RMSEA remained adequate (0.08; Talavera-Velasco et al., 2018).

Another instrument that is widely recognized for measuring FRP is the Job Content Questionnaire (JCQ) was designed to measure job social and psychological characteristics (Karasek et al., 1998). In its original version, it presents five scales: (a) *Psychological demands*, (b) *Decision latitude*, (c) *Social support*, (d) *Physical demands,* and (e) *Job insecurity*. More recent studies have grouped the items into four scales (a) *Demands*: this scale assesses to work very fast, work very hard, and excessive amount of work; (b) *Control*: it assesses enough time to do the job and responds to contradictory orders, many decisions by myself, much freedom to decide how to do the job, and the opinions count a lot; (c) *Job strain:* this scale assesses the job requires that I learn new things, need to be creative, there is variety in the activities to do, and the opportunity to develop the own skills; and (d) *Support:* it assesses the boss or supervisor cares about the economic well-being of the staff in charge, pays attention to what I say, helps to get the job done, and is successful in getting you to work well in a team. The coefficients Cronbach’s alpha ranged between 0.66 and 0.87, and the fit indices (*χ2* = 531.51, df = 3, *p* < 0.001, CFI = 0.93, TLI = 0.92, SRMR = 0.1, RMSEA = 0.09) than the uni-dimensional model (CFI =0.79, TLI = 0.76, SRMR = 0.15, RMSEA = 0.16). In terms of reliability, the demand and the social support subscales showed acceptable reliability (Cronbach’s *α* > 0.7, McDonald’s Omega *ω* > 0.7), but the control subscale displayed questionable reliability (Cronbach’s *α* = 0.61, but McDonald’s *ω* = 0.72; Cerasa et al., 2020).

In Mexico, the questionnaires FPSICO (Contreras, 2011), ISTAS21 (Fernández-Prada et al., 2019), DECORE (Talavera-Velasco et al., 2018), and the *Guide for the Identification of Psychosocial Factors of the Mexican Institute of Social Security* (Aranda et al., 2013) have been used to identify and evaluate the PRF in the workplace. Nevertheless, since 2018, *Official Mexican Standard* NOM-035-STPS-2018 of the Secretary of Labor and Social Security (STPS, for its acronym in Spanish) was published in the Official Journal of the Federation (DOF, for its acronym in Spanish, 2018), which stipulates the use of five *reference guides* for the identification and analysis of PRF and the evaluation of the organizational environment in workplaces (Diario Oficial de la Federación, 2018). It is important to highlight that the design of the *Inventory for the Identification and Analysis of Psychosocial Risk Factors* (IIA-PRF) of the *Reference Guide III* of NOM-035-STPS-2018 is similar to one of the instruments integrated by the battery of instruments for the evaluation of PRF in Colombia, both in the structure and in the methodology for its evaluation (Albarrán et al., 2018). However, a difference is visualized in the wording of the items defined for each factor.

The battery of instruments for evaluating PRF in Colombia comprises five scales (domains) and 20 dimensions, considered factors for its measurement. The first domain associated with (a) *Leadership and social relations at work,* is made up of four dimensions: leadership characteristics, social relations at work, performance feedback, and relationship with collaborators (subordinates). The second domain, (b) *Control over work*, is composed of: role clarity, training, participation and change management, opportunities for the development and use of skills and knowledge, and control and autonomy over work. The third domain, (c) *Job Demands,* is composed of eight dimensions: environmental and physical effort demands, emotional demands, quantitative demands, influence of the job on the extra-work environment, job responsibility demands, mental workload demands, role consistency, and workday demands. The last domain, (d) *Reward,* comprises two dimensions: rewards derived from belonging to the organization and the work performed, and recognition and compensation. Regarding the reliability of each dimension, Cronbach’s reliability coefficients were obtained on average *α* > 0.7. A factor analysis was performed with the principal factor method and oblique Promax rotation, which allowed confirming the statistical grouping of the items whose factor weights were ≥ 0.30 (Ministerio de la Protección Social, 2010).

Few publications have been found in Mexico where the psychometric properties of the IIA-PRF recommended by NOM-035-STPS-2018 are studied. Among the most recent studies are those that evaluate the risk level of workers in the educational, private, and public sectors (Cano, 2020; Cano et al., 2020; Alaniz and López, 2021; Cotonieto-Martínez, 2021). In particular, Cano (2020) conducted a study to determine the domains with the highest level of psychosocial risk and some aspects of the construct validity of the *Reference Guide III* of NOM-035-STPS-2018. The instrument was applied to 1,458 workers (806 women and 652 men) from five workplaces in the industrial sector of the city of Ensenada, Baja California. Among the results, it stands out that: (1) the most frequent domains with medium, high, and very high-risk levels were *Workload*, *Lack of control over work,* and *Workday*; (2) significant differences were found between risk levels (2.31E-20) and in the interaction of risk levels with domains (*p* = 5.06E-36); (3) the instrument presented adequate reliability indexes (Cronbach’s *α* = 0.93, ordinal RHO alpha *ρ* = 0.95, and McDonald’s Omega *ω* = 0.95); (4) the categories presented Cronbach’s *α* coefficient, ordinal RHO alpha *ρ* and McDonald’s Omega *ω* above 0.7, except for the *Work Environment* scale (0.67); and (5) the EFA presented KMO values above 0.66 in all categories and adequate goodness-of-fit indices only for the *Leadership* and *Work Relations* categories (RMSEA 0.072 < 0.08).

Likewise, different researcher studies on the psychometric properties of the *Reference Guide III* of NOM-035-STPS-2018 report unfavorable evidence given the lack of compliance with the criteria established in the same standard (Littlewood-Zimmerman et al., 2020; Uribe et al., 2020). In addition, the studies focus on the same type of activity and are limited to a reliability validation without delving into a proposal of the internal structure of the instrument (Espejel et al., 2022; Gutiérrez et al., 2022).

In particular, Littlewood-Zimmerman et al. (2020) evaluated the reliability and validity of the *Reference Guide III* of NOM-035-STPS-2018, composed of 72 items organized into five categories, 10 domains, and 25 dimensions. They applied the instrument to a sample of 1,247 workers of a gastronomic company in Mexico during September and October 2019. Among their results, they highlight that all categories obtained a Cronbach’s Alpha equal to or greater than 0.70. However, they did not meet the construct validity criteria derived from the Exploratory Factor Analysis (EFA) and the Confirmatory Factor Analysis (CFA), according to numeral 7.5 of NOM-035-STPS-2018. For his part, Uribe et al. (2020) analyzed the psychometric properties of the *Reference Guide III* for a sample of 114 workers from a service company stratified by gender. The analyses included indicators of central tendency and dispersion, reliability calculation using Cronbach’s Alpha, CFA with Structural Equation Modeling (SEM) for validity, Pearson’s correlations, and one-way analysis of variance to know some relationships descriptively. The authors concluded that the STPS instrument has good reliability but no evidence of internal structure validity.

It is important to emphasize that the assessment of PRF in Mexican labor legislation is relatively recent, but their importance at different levels of daily life and in the health of workers in the workplace is unquestionable (Andersen et al., 2022). Nevertheless, these efforts to apply labor policies and integrate action protocols and evaluations on PRF have not achieved satisfactory results, reducing the evaluation of the construct around concepts such as burnout or occupational stress and leaving aside other psychosocial working conditions of an important psychosocial nature for health (Rudkjoebing et al., 2020).

Given the need for valid and reliable instruments to measure PRF, it is important to conduct research focused on the design, development, and evaluation of instruments that provide evidence of the reliability and validity of these processes. Accordingly, this study aims to analyze the internal structure and factorial invariance of the IIA-PRF of workplaces in Mexico’s *industrial* and *education-government* sectors with respect to four categories of PRF. The relevance of having an instrument with an adequate internal structure allows workplaces to evaluate the PRF adequately with reliable results to establish intervention actions. The instrument proposed by NOM-035-STPS-2018 makes no difference for its application in the type of sector or activity of the work center; however, the perception of workers on the PRF in each sector could be different, which would imply avoiding making comparisons on it.

## Materials and methods

### Participants and procedure

Employees of organizations in the *industrial* and *education*-*government* sectors in Mexico were invited to participate in the study. Non-probability sampling with voluntary participation was used. A total of 2,149 workers participated in the study, of which 1,707 (79%) were private sector workers in the *industry* sector, and 442 (21%) were university professors and government employees in the *education-government* sector. For the application of the IIA-PRF, the collaboration and approval of the organizations were requested to conduct the study based on the provisions of the *Mexican Official Standard* NOM-035-STPS-2018. This regulation requires the application to be carried out voluntarily and confidentially; nevertheless, the work centers requested that it be anonymous and thus reduce the possible adverse actions for the workers due to their answers. In addition, the workers who participated voluntarily were required to give their written consent to participate in the study.

The application of the IIA-PRF was in physical and electronic format and was carried out in person and virtually based on the conditions of the workers and their work centers. In the case of the face-to-face application, it was carried out in a space within the workplace free of distractions for the tranquility of the workers. In the case of the virtual application, it was carried out through an electronic platform available on the Internet that made it easier for workers to answer the inventory from any mobile device at the time and place that was most convenient for them. A database cleaning was performed from the data collected, following the recommendations of Hair et al. (2019) and Tabachnick and Fidell (2018). No missing data or erroneous values were found. Cases with outlier scores were identified and removed by visual analysis of a box-and-whisker plot.

### Measurement

For the present study, the psychometric properties of the *Inventory for the Identification and Analysis of Psychosocial Risk Factors* (IIA-PRF) of the *Reference Guide III* proposed in NOM-035-STPS-2018 were analyzed (Diario Oficial de la Federación, 2018). This *Mexican Official Standard* presents five *Reference Guides*: (a) *Reference Guide I* includes a questionnaire that has the purpose of identifying workers who were subjected to severe traumatic events; (b) *Reference Guide II* includes a questionnaire that has the purpose of identifying and analyzing psychosocial risk factors; (c) *Reference Guide III* includes a questionnaire that has the purpose of identifying and analyzing psychosocial risk factors and evaluating the organizational environment in workplaces; (d) *Reference Guide IV* presents an example of a psychosocial risk prevention policy that is intended to be a complement for a better understanding of the Standard; and (e) *Reference Guide V* presents a questionnaire to collect sociodemographic and work data from employees.

In particular, *Reference Guide III* establishes two instruments recommended for evaluating the organizational climate in work centers with a population of more than 50 workers (see Table 1): the IIA-PRF and the *Scale for the Evaluation of the Organizational Environment in Workplaces* (SEOEW). Specifically, the IIA-PRF is composed of 62 Likert-type items distributed in seven factors ([F1] *Work environment conditions*, [F2] *Workload*, [F3] *Lack of control over work*, [F4] *Work schedule*, [F5] *Interference in the work-family relationship*, [F6] *Leadership*, [F7] *Relationships at work*, and [F8] *Violence*) with response options ranging from *Never* = 1 to *Always* = 5. At a second level, the scales are organized into four categories: (a) *Work environment*, (b) *Activity-specific factors*, (c) *Work organization*, and (d) *Leadership and labor relations*. Note that the structure of the IIA-PRF presents three levels of disaggregation and that how it organizes the operationalization of the construct goes against several of the assumptions to correctly delimit its dimensionality from a nomological approach (Cronbach, 1951; Jöreskog and Sörbom, 1979; Hu and Bentler, 1999; Thielmann and Hilbig, 2019). Also, it is important to point out that F4 (*Hours of work*) does not have the minimum number of items necessary for its representation (Hair et al., 2019). It is important to mention that authors such as Patlán-Pérez (2019) highlight that NOM-035-STPS-2018 presents multiple incongruities and inconsistencies in form and format, in addition to lacking a theoretical model and presenting methodological weaknesses for the identification, analysis, evaluation, and prevention of PRF. For more information on the items, structure, and criteria of the IIA-PRF, it is recommended to review the official page of the *Mexican Official Standard* NOM-035-STPS-2018 (Diario Oficial de la Federación, 2018).

**Table 1:** Structure of the two instruments of the Reference Guide III recommended by NOM-035-STPS-2018 for the evaluation of the organizational climate in workplaces with a population of more than 50 workers.

Instruments	Categories	(Factors) Domains	Dimensions	Item position
Inventory for the Identification and Analysis of Psychosocial Risk Factors (IIA-PRF) (k = 62)	1.1. Working environment (k = 5)	(F1) Conditions in the working environment	Hazardous conditions	1, 3
			Substandard and unsanitary conditions	2, 4
			Hazardous work	5
	1.2. Activity-specific factors (k = 35)	(F2) Workload (k = 15)	Quantitative loads	6, 12
			Accelerated working rhythms	7, 8
			Mental load	9, 10, 11
			Emotional psychological loads	65, 66, 67, 68
			Loads of high responsibility	13, 14
			Contradictory or inconsistent loads	15, 16
		(F3) Lack of control overwork (k = 10)	Lack of control and autonomy overwork	25, 26, 27, 28
			Limited or no possibility of development	23, 24
			Insufficient participation and change management	29, 30
			Limited or no training	35, 36
	1.3. Organization of working time (k = 6)	(F4) Working hours (k = 2)	Long working hours	17, 18
		(F5) Interference in the work-family relationship (k = 4)	Influence of work outside the workplace	19, 20
			Influence of family responsibilities	21, 22
	1.4. Leadership and relationships at work (k = 26)	(F6) Leadership (k = 9)	Lack of clarity of roles	31, 32, 33, 34
			Characteristics of Leadership	37, 38, 39, 40, 41
		(F7) Relationships at work (k = 9)	Social relations at work	42, 43, 44, 45, 46
			Poor relationship with the employees he/she supervises	69, 70, 71, 72
		(F8) Violence (k = 8)	Workplace violence	57, 58, 59, 60, 61, 62, 63, 64
Scale for the Evaluation of the Organizational Environment in Workplaces (SEOEW) (k = 10)	2.1. Organizational environment	Recognition of performance (k = 6)	Little or no performance feedback	47, 48
			Little or no recognition and compensation	49, 50, 51, 52
		Insufficient sense of belonging and instability (k = 4)	Limited sense of belonging	55, 56
			Job instability	53, 54

Factor F1 is derived from subsection 7.2.a of NOM-035-STPS-2018, F2 of 7.2.b, F3 of 7.2.c, F4 of 7.2.d, F5 of 7.2.e, F6 of 7.2.f.1, F7 of 7.2.f.2, and F8 of 7.2.g. Adapted from: (Diario Oficial de la Federación, 2018).

### Data analysis

This section was organized in three stages: (1) preliminary analyzes and reliability, (2) obtaining evidence of construct validity of the internal structure, and (3) obtaining evidence of factor invariance according to the *origin of the activity* of each company. During the first stage, descriptive statistics were obtained: mean, standard deviation, skewness, kurtosis, item-total score correlation (*rpbis*), and the estimation of the risk levels according to the overall rating criterion established in section III.3 of NOM-035-STPS-2018 (Diario Oficial de la Federación, 2018) were calculated. Subsequently, the assumptions of multivariate normality, Sample Size Adequacy (SSA), and internal consistency of the test scores were verified.

The criteria for the acceptance of adequate item-total score correlation was fixed at a value *rpbis* ≥ 0.20 (Brown, 2011). Likewise, the multivariate normality assumption was analyzed through the multivariate normality test of skewness and kurtosis of Mardia (1970), and the criterion for acceptance of multivariate normality in the sample was a statistically non-significant value (*p* ≥ 0.05). The assumption of sample adequacy was checked through Bartlett’s test of sphericity and the Kaiser-Meyer-Olkin (KMO) coefficient, the criterion for acceptance of the assumption was a *value of p* ≤ 0.50 in Bartlett’s test and ≥0.70 for the KMO coefficient (Hair et al., 2019). For its part, the internal consistency assumption was evaluated through the calculation of Cronbach’s index (*α*), Rho’s standardized ordinal Alpha (*ρ*), and McDonald’s Omega coefficient (*ω*), the criteria for the acceptance of the assumption were the obtaining of values *α* ≥ 0.70, *ρ* ≥ 0.70 and *ω* ≥ 0.80 (Cronbach, 1951; Zhang and Yuan, 2016; JonasMoss, 2020).

In the second stage, a series of analyses were carried out to obtain evidence of the construct validity of the internal structure aspect. First, a CFA with Diagonally Weighted Least Squares (DWLS) estimation (Brown, 2015; Kline, 2015) was implemented in two models suggested by NOM-035-STPS-2018 (Diario Oficial de la Federación, 2018). According to said normativity, PRF can be analyzed at three latent structural levels defined by categories, domains, and dimensions. Considering the recommendations of said standard and the advances of substantive research in the delimitation of the construct, two models were analyzed: a first model (8-FM) aligned at the domain level (*Work environment conditions* [F1], *Workload* [F2], *Lack of control over work* [F3], *Work schedule* [F4], *Work-family relationship interference* [F5], *Leadership* [F6], *Work relationships* [F7], and *Violence* [F8]) and a second model (4-FM) aligned at the category level (*Work environment* [F1], *Activity-specific factors* [F2], *Work time organization* [F3], and *Leadership and work relationships* [F4]). The fit of the analyzed models was evaluated following the suggestions of Hu and Bentler (1999) and Hair et al. (2019). The fit indices and criteria are CFI ≥ 0.95, Tucker-Lewis Index (TLI) ≥ 0.95, Standardized Root Mean Square Residual (SRMR) ≤ 0.08, and RMSEA ≤0.06 (Browne and Cudeck, 1993; Schreiber et al., 2006). Second, the degree of correlation between factors was calculated to verify whether they present coherent relationships or correspondence (De Winter et al., 2016; Liddell and Kruschke, 2018). NOM-035-STPS-2018 (Diario Oficial de la Federación, 2018) indicates that an instrument with adequate evidence of correlation must present significant correlation coefficient values and with a value equal to or greater than 0.50 (r ≥ 0.50), so this criterion will be followed.

Because both models (8-FM and 4-FM) suggested by NOM-035-STPS-2018 explain only 34 and 27% of the total variance, which is less than the specialists’ recommendation (Tabachnick and Fidell, 2018) and did not meet the criteria of a good fit, it was decided to reconfigure the IIA-PRF. To do this, based on the recommendations of three PRF measurement specialists, the modification indexes, the standardized factor loadings, and the error variances were considered, and the items that did not meet the quality criteria were eliminated. As a result, the specialists proposed a three-factor model that only includes items that meet the quality and fit criteria in the factorial model (see Table 2). The proposed three scales refer to: (a) *Conflicting relationships with co-workers and managers* (F1), (b) *Negative effects of working conditions on personal and family life and work relations* (F2), (c) *Mental and quantitative burdens and accelerated work rhythms* (F3). Once the 3-FM was reconfigured, it was complemented with the diagonally weighted least squares model for the CFA following the suggestions of Hu and Bentler (1999) for the evaluation of model fit. In total, three models were analyzed: (1) a model of eight factors (8-FM) aligned to the domains proposed by NOM-035-STPS-2018; (2) a model of four factors (4-FM) aligned with the categories established by the same standard; and (3) a model of three factors (3-FM) adjusted based on the results of a first study of the psychometric properties of the IIA-PRF and the recommendations of specialists in the field. All cases (N = 2,149) were used to obtain the fit indices for 8-FM, and for models 4-FM and 3-FM, the sample was to 1,500 randomly selected cases.

**Table 2:** Factors and items of the 8-FM, 4-FM, and 3-FM models of the IIA-PRF.

Model	Factors	Item position (Q)
8-FM (k = 62)	F1 Conditions in the working environment (k = 5)	1, 2, 3, 4, and 5
	F2 Workload (k = 15)	6, 7, 8, 9, 10, 11, 12, 13, 14, 15, 16, 65, 66, 67, and 68
	F3 Lack of control overwork (k = 10)	23, 24, 25, 26, 27, 28, 29, 30, 35, and 36
	F4 Working hours (k = 2)	17 and 18
	F5 Interference in the work-family relationship (k = 4)	19, 20, 21, and 22
	F6 Leadership (k = 9)	31, 32, 33, 34, 37, 38, 39, 40, and 41
	F7 Relationships at work (k = 9)	42, 43, 44, 45, 46, 69, 70, 71, and 72
	F8 Violence (k = 8)	57, 58, 59, 60, 61, 62, 63, and 64
4-FM (k = 51)	F1 Working environment (k = 5)	1, 2, 3, 4, and 5
	F2 Activity-specific factors (k = 25)	6, 7, 8, 11, 15, 16, 65, 66, 67, 68, 23, 24, 25, 27, 29, 30, 35, and 36
	F3 Organization of working time (k = 6)	17, 18, 19, 20, 21, and 22
	F4 Leadership and relationships at work (k = 26)	31, 32, 33, 34, 37, 38, 39, 40, 41, 42, 43, 44, 45, 46, 57, 58, 59, 60, 61, 62, 63, and 64
3-FM (k = 42)	F1 Conflicting relationship with co-workers and managers (k = 20)	4, 23, 24, 30, 31, 32, 33, 34, 35, 36, 37, 38, 39, 40, 41, 42, 43, 44, 45, and 46
	F2 Negative effects of working conditions on personal and family life and work relations (k = 19)	2, 3, 5, 15, 16, 17, 18, 19, 20, 21, 22, 29, 58, 59, 60, 61, 62, 63, and 64
	F3 Mental and quantitative burdens and accelerated work rhythms (k = 3)	6, 7, and 11

Finally, in the third stage, to obtain evidence of factor invariance, a Multigroup Confirmatory Factor Analysis (MFCMG) based on the structure of the 3-FM model was performed. The invariance assumption was verified according to the *origin of the activity* of each company; for this purpose, two denominations were considered: organizations in the *industrial* and *education-government* sectors. Models were compared to obtain evidence of *configurational, metric, scalar,* and *error invariance* (Dimitrov, 2010; Milfont and Fischer, 2010) because the value of the Chi-square statistic is sensitive to sample size (Vandenberg and Lance, 2000; Cheung and Rensvold, 2002); a CFI difference of less than −0.01 (ΔCFI ≤ 0.01) and an RMSEA difference of less than 0.015 (ΔRMSEA ≤ 0.01) were set as criteria (Chen, 2007; Dimitrov, 2010; Putnick and Bornstein, 2016). It is important to note that according to Meade and Lautenschlager (2004) and Meade (2005), for sample sizes ≥ 400 per subgroup, there is a 50% to 100% chance that the metric invariance test is significant.

Preliminary statistical analyses, as well as CFA and MFCMG following the recommendations of Hirschfeld and Von-Brachel (2014), Hu and Bentler (1999), and Hair et al. (2019), were performed with the open source software RStudio version 1.4 (Team, 2022) via the packages dplyr (Wickham et al., 2019), psych (Revelle and Revelle, 2015), lavaan (Rosseel, 2012) and semTools (Jorgensen et al., 2022). In addition, preliminary analyses were accompanied using IBM SPSS version 20.0 (SPSS Inc., Chicago, IL).

## Results

### Results of the preliminary analyzes and reliability

Table 3 shows the mean, standard deviation, skewness, kurtosis, and item-total score correlation values for the 62 items of *Reference Guide III* recommended by NOM-035-STPS-2018. The averages of the 5-point Likert scale items were relatively above the mean, with values ranging from 1.52 (Q1) to 4.58 (Q72). The multivariate normality test of skewness and kurtosis of Mardia (1970) obtained significant results (*p* < 0.001), rejecting the assumption of multivariate normality in the sample studied. In particular, items Q9, Q10, Q12, Q13, Q14, Q26, Q28, Q69, Q70, Q71, and Q72 did not meet the criteria for acceptance of an adequate item-to-total score correlation, which was set at a *rpbis* value ≥ 0.20 (Brown, 2011). Likewise, the result of the Sample Size Adequacy (SSA = 0.95) allows us to assume that the variables are related to each other.

**Table 3:** Descriptive statistics of the General Index (GI) of the IIA-PRF and by item.

(Factors) Domains	Item position	Mean	Standard deviation	Asymmetry	Kurtosis	*rp*BIS
(F1) Conditions in the working environment	Q1	1.52	0.82	1.79	3.43	0.33
	Q2	3.09	1.21	−0.09	−0.77	0.44
	Q3	3.02	1.41	−0.09	−1.25	0.36
	Q4	1.73	0.98	1.36	1.37	0.40
	Q5	3.36	1.46	−0.39	−1.2	0.48
(F2) Workload	Q6	3.45	1.06	−0.18	−0.4	0.33
	Q7	3.15	1.15	−0.09	−0.6	0.34
	Q8	2.99	1.12	0.06	−0.44	0.21
	Q9	2.2	1.37	0.85	−0.6	−0.34*
	Q10	2.8	1.16	0.11	−0.66	0.07*
	Q11	3.2	1.15	−0.09	−0.65	0.32
	Q12	3.25	1.23	−0.14	−0.86	0.16*
	Q13	2.68	1.48	0.30	−1.29	0.02*
	Q14	2.75	1.60	0.26	−1.50	−0.06*
	Q15	3.41	1.27	−0.39	−0.78	0.56
	Q16	3.64	1.29	−0.61	−0.65	0.61
	Q65	4.31	1.13	−1.50	1.15	0.21
	Q66	4.33	1.29	−1.72	1.45	0.27
	Q67	4.29	1.29	−1.63	1.23	0.24
	Q68	4.38	1.33	−1.88	1.85	0.28
(F3) Lack of control overwork	Q23	2.43	1.09	0.30	−0.47	0.34
	Q24	2.81	1.32	0.07	−1.04	0.50
	Q25	2.87	1.10	0.07	−0.41	0.21
	Q26	3.4	1.26	−0.26	−0.95	0.08*
	Q27	2.82	1.19	0.22	−0.67	0.21
	Q28	3.34	1.24	−0.08	−0.95	−0.01*
	Q29	3.48	1.12	−0.32	−0.47	0.42
	Q30	3.19	1.22	−0.07	−0.79	0.33
	Q35	2.17	1.28	0.79	−0.47	0.47
	Q36	2.29	1.27	0.61	−0.68	0.57
(F4) Working hours	Q17	3.59	1.28	−0.55	−0.7	0.41
	Q18	3.28	1.29	−0.22	−0.91	0.45
(F5) Interference in the work-family relationship	Q19	3.49	1.29	−0.44	−0.81	0.54
	Q20	3.97	1.27	−0.93	−0.32	0.39
	Q21	3.35	1.14	−0.19	−0.56	0.40
	Q22	3.81	1.42	−0.89	−0.61	0.50
(F6) Leadership	Q31	1.91	1.05	1.04	0.48	1.91
	Q32	1.93	1.08	1.02	0.33	1.93
	Q33	1.88	1.06	1.11	0.58	1.88
	Q34	1.91	1.07	1.04	0.37	1.91
	Q37	2.17	1.28	0.79	−0.47	2.17
	Q38	2.29	1.27	0.61	−0.68	2.29
	Q39	2.38	1.20	0.54	−0.52	2.38
	Q40	2.47	1.22	0.46	−0.64	2.47
	Q41	2.29	1.17	0.63	−0.4	2.29
(F7) Relationships at work	Q42	2.34	1.08	0.56	−0.14	0.37
	Q43	2.02	1.06	0.90	0.26	0.44
	Q44	1.94	1.04	0.99	0.44	0.50
	Q45	1.95	0.98	0.88	0.38	0.47
	Q46	2.04	1.06	0.79	−0.01	0.45
	Q69	4.56	0.98	−2.26	4.24	0.05*
	Q70	4.57	1.01	−2.41	4.75	0.07*
	Q71	4.56	1.05	−2.34	4.31	0.04*
	Q72	4.58	1.02	−2.45	4.89	0.07*
(F8) Violence	Q57	2.25	1.14	0.69	−0.16	0.39
	Q58	3.4	1.29	−0.38	−0.89	0.51
	Q59	3.8	1.49	−0.87	−0.75	0.56
	Q60	3.77	1.41	−0.8	−0.72	0.55
	Q61	3.78	1.50	−0.84	−0.8	0.60
	P62	3.74	1.46	−0.76	−0.84	0.59
	Q63	3.72	1.49	−0.74	−0.93	0.59
	Q64	3.93	1.51	−1.04	−0.51	0.53
Average		3.06	1.22	−0.14	0.01	0.72
GI		204.15	37.23	−0.54	1.24	

* Items not meeting criteria rpbis ≥ 0.20.

### Internal structure

The results of the internal consistency indices allow us to verify the assumption of internal consistency of each of the models analyzed. The overall internal consistency indices for 8-FM yielded values *α* = 0.93, *ρ* = 0.93, and *ω* = 0.94; for 4-FM, *α* = 0.90, *ρ* = 0.90, and *ω* = 0.95; and for 3-FM, *α* = 0.90, *ρ* = 0.91, and *ω* = 0.92. All the models at the factor level presented adequate internal consistency values according to the criteria established *a-priori* except F1 (*α* = 0.67 and *ρ* = 0.65) and F4 (*α* = 0.67 and *ρ* = 0.67) of 8-FM and F1 (*α* = 0.55 and *ρ* = 0.48) of 4-FM. The 3-FM presented adequate values in all the fit indices, being the one that best represents the empirical data in an underlying model (see Table 4).

**Table 4:** Internal consistency indices for the factors and global instrument of the IIA-PRF provided by the STPS.

Models	Factors	Internal consistency indexes		
		*Cronbach’s index (α)*	*Standardized Rho alpha (ρ)*	*McDonald’s Omega coefficient (ω)*
Eight-factor model (8-FM) aligned to the categories proposed by NOM-035-STPS-2018 (k = 62)	F1 (k = 5)	0.67*	0.65*	0.79
	F2 (k = 15)	0.77	0.78	0.88
	F3 (k = 10)	0.76	0.76	0.84
	F4 (k = 2)	0.67*	0.67*	-
	F5 (k = 4)	0.81	0.81	0.85
	F6 (k = 9)	0.93	0.93	0.92
	F7 (k = 9)	0.81	0.81	0.88
	F8 (k = 8)	0.92	0.91	0.90
Global		0.93	0.93	0.94
Four-factor model (4-FM) aligned to the domains proposed by NOM-035-STPS-2018 (k = 51)	F1 (k = 5)	0.55*	0.48*	0.79
	F2 (k = 18)	0.77	0.77	0.88
	F3 (k = 6)	0.85	0.85	0.89
	F4 (k = 22)	0.86	0.86	0.88
Global		0.90	0.90	0.95
Adjusted three-factor model (3-FM) (k = 42)	F1 (k = 20)	0.94	0.94	0.95
	F2 (k = 19)	0.95	0.95	0.96
	F3 (k = 3)	0.63	0.63	0.64
Global		0.90	0.90	0.93

Reliability criteria α ≥ 0.70, ρ ≥ 0.70 and ω ≥ 0.80.

* Results that do not meet the quality criteria established *a priori.*

Concerning the CFA, the fit was evaluated for each of the underlying IIA-PRF models. Table 5 shows that the 8-FM and 4-FM models have low indices of adequate fit values. The 3-FM model, nevertheless, presents adequate values in all the fit indices.

**Table 5:** Values of fit indexes in different underlying models of the IIA-PRF.

Model	*χ* ^2^	*gl*	*p*	CFI	NNFI	GFI	SRMR	RMSEA
8-FM	28538.5	1801	<0.01	0.77	0.76	0.83	0.13	0.10
4-FM	29323.9	1,424	<0.01	0.69	0.67	0.76	0.16	0.14
3-FM	11683.4	1762	<0.01	0.95	0.95	0.96	0.07	0.06

χ^2^, Chi-square; gl, degrees of freedom; *p*, significant value; CFI, Comparative Fit Index; NNFI, Non-Normed Fit Index; GFI, Goodness-of-Fit Index; SRMR, Square Root Mean Residual; RMSEA, Root Mean Square Error of Approximation.

It is important to remember that for the estimation of the 3-FM, items that did not meet the criteria of *rpbis* value ≥ 0.20 and factor loadings *λ* ≥ 0.43 were eliminated (Q1, Q8, Q9, Q10, Q12, Q13, Q14, Q25, Q26, Q27, Q28, Q57, Q65, Q66, Q67, Q68, Q69, Q70, Q71, and Q72). The average of the 8-FM inter-factor correlations is 0.36, with a range between 0.03 and 0.68. All correlation coefficients were positive and statistically significant (*p* < 0.001); however, only five correlations between factors (F2-F5, F3-F6, F4-F5, F5-F8, and F7-F8) present values above the criterion established in NOM-035-STPS-2018 (*r* ≥ 0.50; Diario Oficial de la Federación, 2018).

Table 6 shows the 3-FM of the IIA-PRF proposed by the specialists based on the modification indexes, the standardized factor loadings and the error variances were considered, and the items that did not meet the quality criteria were eliminated. The F1 relating to *Conflictive relationships with colleagues and manager*s consists of 20 items measuring little or no possibility of development, little or no training, lack of clarity of roles, type of leadership of the boss, the consideration by managers of ideas and proposals from employees, and quality of social relations at work. The F2 associated with the *Negative effects of working conditions on personal and family life and work relationships* consists of 19 items measuring the level of violence at work, long working hours, influence of work outside the workplace, untimely changes affecting work, contradictory or inconsistent workload, attention from angry customers, and dangerous, poor, and unhealthy working conditions. The F3 measures *Mental and quantitative burdens and accelerated work rhythms* with three items. The factor loadings of the F1 items present values from 0.46 to 0.078, the F2 items from 0.50 to 0.86, and the F3 items ranging from 0.55 to 0.66.

**Table 6:** Standardized factor loadings for the adjusted three-factor model (3-FM) of the IIA-PRF.

F1 Conflicting relationship with co-workers and managers (k = 20)		F2 Negative effects of working conditions on personal and family life and work relations (k = 19)		F3 Mental and quantitative burdens and accelerated work rhythms (k = 3)	
Q4	0.50	Q2	0.58	Q6	0.66
Q23	0.46	Q3	0.50	Q7	0.55
Q24	0.56	Q5	0.70	Q11	0.61
Q30	0.48	Q15	0.74		
Q31	0.71	Q16	0.80		
Q32	0.72	Q17	0.58		
Q33	0.74	Q18	0.64		
Q34	0.74	Q19	0.69		
Q35	0.63	Q20	0.65		
Q36	0.70	Q21	0.55		
Q37	0.75	Q22	0.77		
Q38	0.72	Q29	0.60		
Q39	0.75	Q58	0.71		
Q40	0.78	Q59	0.84		
Q41	0.72	Q60	0.78		
Q42	0.52	Q61	0.86		
Q43	0.59	Q62	0.85		
Q44	0.68	Q63	0.82		
Q45	0.63	Q64	0.81		
Q46	0.59				

Criteria of factor loadings λ ≥ 0.43.

### Measurement invariance

Subsequently, an MFCMG was applied to verify whether the conceptualization of the PRF model is the same among workers in different sectors. The 3-FM was considered the basis for the analysis due to its adequate and superior fit compared to the other models. As shown in Table 7, for the most part, the models present adequate fit indices for workers in the *industrial* and *education-government* sectors; therefore, it is inferred that the structure or dimensionality of the model is invariant between both groups of workers. The cases of workers in the *higher education* sector were not considered for invariance measurement because they represent a small number (N = 98) that may bias comparisons, and the fit indices for this population were below the criteria established *a-priori* (Meade and Lautenschlager, 2004; Meade, 2005).

**Table 7:** Fit values for the configurational invariance model as a function of different sectors of activity.

Origin of the activity	*χ^2^*	*gl*	*p*	CFI	NNFI	GFI	SRMR	RMSEA
Industrial sector	5839.56	816	<0.01	0.94	0.9^*^	0.95^*^	0.07^*^	0.06^*^
Education-government sector	1707.23	816	<0.01	0.97^*^	0.96^*^	0.95^*^	0.09	0.06^*^

χ^2^, Chi-square; gl, degrees of freedom; SB/χ^2^, Chi-square of Satorra-Bentler; CFI, Comparative Fit Index; NNFI, Non-Normed Fit Index; GFI, Goodness-of-Fit Index; SRMR, Square Root Mean Residual; RMSEA, Root Mean Square Error of Approximation.

* Value suggesting adequate model fit.

The indices obtained in the configurational invariance model suggest evidence of poor fit as a function of the *origin of the activity*. As shown in Table 8, the CFI and Non-Normed Fit Index (NNFI) values are below 0.95, and the RMSEA and SRMR values are above 0.06 and 0.08, respectively. However, evidence of metric invariance meets the criteria established *a-priori* (ΔCFI ≤ 0.01 and ΔRMSEA ≤ 0.01), the *metric invariance* assumption is rejected, indicating that the hypothesized model does not support the equivalence of factor loadings in both groups. As a result, it is not advisable to carry out score comparison analyses between groups based on the origin of the activity.

**Table 8:** Fit index values for obtaining evidence of factorial invariance as a function of the origin of the activity.

Model	*χ* ^2^	*gl*	CFI	ΔCFI	NNFI	RMSEA	ΔRMSEA	SRMR
Configural invariance	15806.60	1,632	0.69	-	0.67	0.09	-	0.07
Metric invariance	16009.11	1,671	0.69	−0.004*	0.68	0.09	−0.001*	0.08
Scalar invariance	18119.91	1710	0.64	−0.045	0.64	0.10	0.005*	0.11
Error invariance	18646.01	1752	0.63	−0.011	0.64	0.10	0.000*	0.11

χ^2^, Chi-square; gl, degrees of freedom; CFI, Comparative Fit Index; ΔCFI, difference between CFI values; RMSEA, Root Mean Square Error of Approximation; ΔRMSEA, difference between RMSEA values.

* Value suggesting adequate model fit.

## Discussion

The detection and analysis of PRF at work are one of the most important aspects of safeguarding workers’ physical, social and mental well-being (Burr, 2021). In Mexico, the *Reference Guide III* of NOM-035-STPS-2018 was developed to identify, analyze and prevent PRF in workplaces. This *Mexican Official Standard* recommends using *ad hoc* instruments to identify and analyze PRF and evaluate a favorable organizational environment (Diario Oficial de la Federación, 2018). In the present research, the psychometric properties and validity evidence of the internal structure of the IIA-PRF were evaluated based on the analysis of the domains (8-FM) and categories (4-FM) established in NOM-035-STPS-2018. In addition, a three-factor model (3-FM) that meets the reliability and dimensionality criteria is proposed.

In particular, evidence of the technical quality of three models (8-FM, 4-FM, and 3-FM) generated from the IIA-PRF is presented. First, a notable number of items of the IIA-PRF (k = 11) show inadequate discrimination values (*rpbis* < 0.20), therefore, its revision, based on the recommendation of specialists and verification with consolidated theoretical and measurement models, is recommended for its improvement. Second, the internal consistency indices of the IIA-PRF present adequate overall values in the three models analyzed (8-FM [*α* = 0.93; *ρ* = 0.93; *ω* = 0.94], 4-FM [*α* = 0.90; *ρ* = 0.90; *ω* = 0.95], and 3-FM [*α* = 0.90; *ρ* = 0.90; *ω* = 0.93]). Regarding the internal consistency at the factor level, in the 8-FM model, only F1 (*α* = 0.67, *ρ* = 0.65, *ω* = 0.79), F4 (*α* = 0.67, *ρ* = 0.67, *ω* = −), and the F1 factor (*α* = 0.55, *ρ* = 0.48, *ω* = 0.79) of the 4-FM do not meet the quality standards for internal consistency. On the other hand, all the factors of the 3-FM meet the reliability criteria (*α* ≥ 0.70, *ρ* ≥ 0.70, *ω* ≥ 0.80). These results agree with the findings by Uribe et al. (2020), who presented adequate internal consistency values of the IIA-PRF in a model similar to the 8-FM. However, at the factor level, F1 obtained an *α* = 0.68 and F4 an *α* = 0.69. Likewise, Littlewood-Zimmerman et al. (2020) conduct a study where they model a 4-factor structure at the IIA-PRF category level. Their findings show that F1 presents *α* = 0.739, unlike the other three factors with *α* values > 0.80.

Third, the 8-FM model of the IIA-PRF proposed in *Reference Guide III* did not show evidence of a good fit. These results agree with the results obtained by Uribe et al. (2020) and Cano et al. (2020). The fit indices presented lower values (NFI = 0.087, CFI = 0.94, RMSEA = 0.154) than the established criteria. The 4-FM model also did not reach the expected fit indices (CFI = 0.462, NFI = 0.897, SRMR = 0.874, RMSEA = 0.138). Similarly, these results agree with the findings presented by Littlewood-Zimmerman et al. (2020). For its part, the three-factor model (3-FM) proposed by the specialists presents acceptable values of internal consistency (*α* = 0.90; *ρ* = 0.91; *ω* = 0.92) and adequate fit indices (CFI = 0.95, NFI = 0.96, SRMR = 0.07, RMSEA = 0.07). The factor loadings of the items that make up the 3-FM meet the criterion *λ* ≥ 0.43. It is important to note that the 3-FM meets the quality and fit criteria established in NOM-035-STPS-2018 (CFI > 0.90, NNFI > 0.90, SRMR < 0.08, RMSEA < 0.08) and that they agree with the psychometric properties of other consolidated instruments to measure PRFs (Talavera-Velasco et al., 2018; Useche et al., 2019; Cerasa et al., 2020). However, it is important to highlight that the F3 of the 3-FM meets the minimum acceptable three-item threshold (Streiner, 1994). Likewise, its use is not recommended for strong decision-making in workplaces because, so far, it does not present substantive theoretical bases to support the interpretation of its results. In addition, it is recommended to take with caution the use of the 3-FM model for the design of work environment improvement programs because there is no conceptualization of a strong granular model based on empirical evidence that meets all the corresponding quality criteria which support the different dimensions of the PRF established in NOM-035-STPS-2018. It is important to remember that the objective of the exercise was to propose a model that complies with the criteria established in the *Mexican Official Standard*.

On the other hand, it is important to highlight the problem that the conceptualization and dimensionality of PRF measurement models, such as the case of the IIA-PRF, still present today (Patlán-Pérez, 2019; Duarte and Vega, 2021). Some authors have analyzed different instruments to measure PRF and, as part of their results, propose models with different numbers of factors (from seven to 24; Moncada et al., 2005; Ministerio de la Protección Social, 2010; Ferrari et al., 2016; Pando et al., 2016). However, the consolidated measurement models that are widely accepted in the field and that present a strong rationale and empirical evidence of their technical quality are those with five factors or less (e.g., COPSOQ [5 factors], JCQ [5 factors], DECORE [4 factors]), and that each of their factors has a considerable number of items exceeding the minimum acceptable (Streiner, 1994). In the case of the IIA-PRF recommended for its application in NOM-035-STPS-2018, it does not take up these consolidated models and does not justify its alignment to the *Battery of Instruments for the Assessment of Psychosocial Risk Factors* developed by the Colombian Ministry of Social Protection (MPS) in 2010 (Ministerio de la Protección Social, 2010), which, in different studies of its psychometric properties does not meet many of the technical quality standards (Rubio-Castro and Luna-García, 2015; Gómez et al., 2016; Albarrán et al., 2018).

As for the measurement of invariability, there are no antecedents to date that report information associated with the IIA-PRF. In the present study, a first effort was made to verify whether the conceptualization of the PRF model is the same among workers in the *industrial* and *education-government* sectors. Although the invariance criteria were met in the different groups based on the difference in indexes, the CFI and RMSEA values suggest that the model does not fit adequately, so the invariance results should be considered cautiously. In particular, for measuring invariance, there were limitations associated with the inclusion criteria, given that the work centers requested anonymity and confidentiality in the data provided by the workers who participated in the study, which reduced the possibility of having other context variables with which to verify whether the conceptualization of the PRF model for the different groups could be verified.

Likewise, a point to highlight is that the data for the group belonging to the higher education sector presented evidence of poor fit in all the models evaluated. Currently, no research delves into the differences between different origins of the activity within Mexico and other relevant variables such as differences by sex or age, job type, and position, so it is recommended to investigate the subject in depth. In summary, it can be said that the evidence of factorial invariance obtained does not allow us to verify that the different groups of participants conceptualize the construct underlying the IIA-PRF in the same way. Although evidence of invariance was obtained in the configurational and metric models, it is not sufficient to make valid comparisons between the participants’ mean scores (Dimitrov, 2010; Milfont and Fischer, 2010).

## Conclusion

It can be said that the IIA-PRF of the *Reference Guide III* of NOM-035-STPS-2018 presents items that do not meet the *rpbis* criterion. Likewise, the 8-FM presents inadequate adjustment values. Also, the 4-FM, even eliminating the items that do not comply with the *rpbis*, presents inadequate adjustment values, as does the 8-FM. On the other hand, the 3-FM proposed by the specialists meets the quality criteria of reliability and factorial structure, so the measurement of the PRF using this model can be supported. However, the 3-FM measurement of PRF is not invariant to the origin of the activity.

Given the results of the invariance measurement models, it is not recommended to perform comparative studies based on the mean scores obtained from IIA-PRF. Finally, expert review and adjustment of the internal structure and item design of the IIA-PRF is recommended, as well as exploring internal structure validity tests using other models such as Item Response Theory (IRT) or Bayesian estimates. In addition, measurement invariance across employees by age, gender, and position is recommended.

Finally, for future research, it is recommended that the IIA-PRF be revised based on consolidated constructs. In turn, it should address NOM-035-STPS-2018 to comply with the corresponding legislation to identify and cater to areas of workers’ work environment in Mexico. Also, a sufficiently granular (multidimensional) model is proposed that identifies important areas to attend to in workers, allowing the formulation of attention programs for improving workers and companies in different topics of a favorable organizational environment. Likewise, it is recommended that further studies on the validity of the inventory be carried out, given that in Mexico, no studies have been found on proposals for models that would make it possible to improve the measurement of the PRF in workplaces based on the IIA-PRF.

## Data availability statement

The raw data supporting the conclusions of this article will be made available by the authors, without undue reservation.

## Author contributions

JC-G and JP-M contributed to the idea of research, its conceptualization, implementation, and methodology, was in charge of writing the manuscript, and also contributed to the analysis and interpretation of data, revising the English version, and writing in Frontiers format. DM and BB-B directed the analysis and interpretation of data and contributed to the research’s conceptualization and the manuscript’s writing, and was also in charge of revising the English version and writing in Frontiers format. RL collaborated in the analysis and interpretation of data, supported the search for additional bibliographic information, and reviewed the style of the article. JO-T contributed to the implementation of the methodology and also contributed to the analysis and interpretation of data, the revision of the first version, and writing in Frontiers format. All authors contributed to the article and approved the submitted version.

## Conflict of interest

The authors declare that the research was conducted without any commercial or financial relationships that could be construed as a potential conflict of interest.

## Publisher’s note

All claims expressed in this article are solely those of the authors and do not necessarily represent those of their affiliated organizations, or those of the publisher, the editors and the reviewers. Any product that may be evaluated in this article, or claim that may be made by its manufacturer, is not guaranteed or endorsed by the publisher.
